# Supplementary material for: Phenotype Bias Determines How Natural RNA Structures Occupy the Morphospace of All Possible Shapes
Source: Mol Biol Evol. 2021 Sep 20;39(1):msab280. doi: 10.1093/molbev/msab280 (PMC8763027; doi:10.1093/molbev/msab280)
Supplement: msab280_Supplementary_Data [file msab280_supplementary_data.pdf]

# Supplementary Information for: Phenotype bias determines how RNA structures occupy the morphospace of all possible shapes

Kamaludin Dingle[1], Fatme Ghaddar[1], Petr Šulc[2], Ard A. Louis[3]
[1] *Centre for Applied Mathematics and Bioinformatics, Department of Mathematics and Natural Sciences, Gulf University for Science and Technology, Hawally 32093, Kuwait,*
[2] *School of Molecular Sciences and Center for Molecular Design and Biomimetics at the Biodesign Institute, Arizona State University, Tempe, AZ, USA*
[3] *Rudolf Peierls Centre for Theoretical Physics, University of Oxford, Parks Road, Oxford, OX1 3PU, United Kingdom*

(Dated: September 16, 2021)

### A. $L = 55$ data for levels 1 to 5

In Figures (S1) and (S2) we show plots for the $L = 55$ data using all five coarse-grained abstraction levels of RNAshapes from Giegerich et al. [1]. These figures demonstrate very similar results to those found in the main text for level 3. This qualitative agreement strongly suggests that our main findings are robust to our choice of level. Note that the lowest possible frequencies directly measured in the database are limited by the relatively small number of samples, which affects lower levels of coarse-graining more strongly, because there are more such shapes available. The rank plots in Figure (S1) suggest that as more sequences are added, a wider range of frequencies will be found, improving the correlation at low frequency in Figure (S2).

### B. $L \approx 100$ data from Rfam

To briefly check that our results maintain for a different database, and with secondary structures not obtained solely by folding algorithms, here we study data from the Rfam [2, 3] database.

All RNAs of length 95 to 105 were taken from all available seed sequences of ncRNA families from the Rfam database. Their secondary structures were obtained by aligning to the consensus structure of the seed alignment for respective RNA families. Note that this is different to analysis we performed for the main text, where instead secondary structures were predicted via folding algorithms, using the popular Vienna package.

The total number of sequences obtained were 4309, but a small fraction (ie 185 or 4.3%) of these were discarded because they were invalid secondary structures according to the folding rules used by the shape abstracter. For example, some of the consensus structures contained motifs with a loop of length 1 — ie (.) — which are deemed invalid. The reason we combined data for lengths 95 to 105 (rather than just using $L = 100$) is that there were relatively few sequences and RNA shapes for just $L = 100$, and so by combining data from other lengths close to 100, we obtain better statistics.

Qualitatively similar rank and correlation plots appear when using Rfam data for $L \approx 100$ in Figure (S3) as compared to the correlation plots in the main text. These results provide evidence that the correlations we find between random sampling and the natural RNA are not artefacts of either the database which we have used, nor of the method for obtaining secondary structures.

### C. Effects of GC content

We also examine the effects of altering the GC content of the sequences on the probabilities of RNA shapes. In the natural sequences which we have used, there does not appear to be strong deviations from 50% GC content: the average GC content is lowest at 44% for $L = 55$ and highest at 57% for $L = 85$. Nonetheless, because GC content can affect RNA structures and shapes[4], we here perform some further analysis regarding the role of GC content in our current study.

We performed 1 million samples of $L = 55$ sequences using level 3 abstraction with 50% GC content, ie using $P(G) = P(C) = P(A) = P(T) = 0.25$ at each nucleotide base, which yielded 150 different RNA shapes. A separate 1 million random sample at 70% GC content (ie $P(G) = P(C) = 0.35$ and $P(A) = P(T) = 0.15$), yielded 189 shapes. Finally, another separate random sample with 30% GC content (ie $P(G) = P(C) = 0.15$ and $P(A) = P(T) = 0.35$) yielded only 82 shapes.

In Figure (S4) we provide correlation plots showing the probability of abstract shapes under different GC content values, namely 50% GC content vs 30% in (a) and 50% GC content vs 70% in (b). The linear correlations were very high: $r = 0.95$ for 30% (p-val$< 10^{-6}$) and $r = 0.97$ for 70% (p-val$< 10^{-6}$). The main effect of the CG content is that neutral set sizes (NSS) are larger for lower CG content, a result that was also obtained in the supplementary materials of [5]. This effect explains why under a fixed number of samples, more structures are observed for higher CG content. However, the relative frequencies are quite similar.

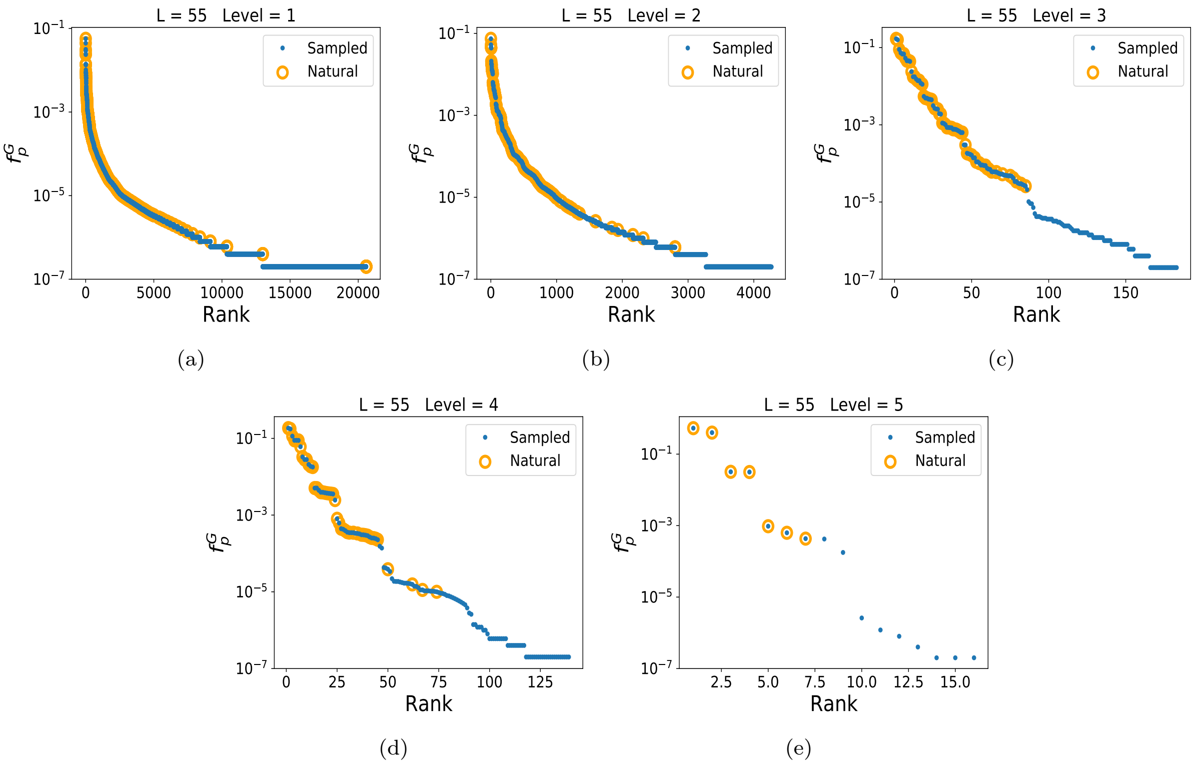


FIG. S1. Rank plot for $L = 55$, across all abstraction levels 1, 2, 3, 4 and 5, with $5 \times 10^6$ random samples for each level, compared to the natural frequencies from the RNAcentral database. The number of random shapes and number of natural shapes (in brackets) found for levels 1—5 are 20587 (1083), 4268 (394), 183 (63), 139 (46), and 16 (7).

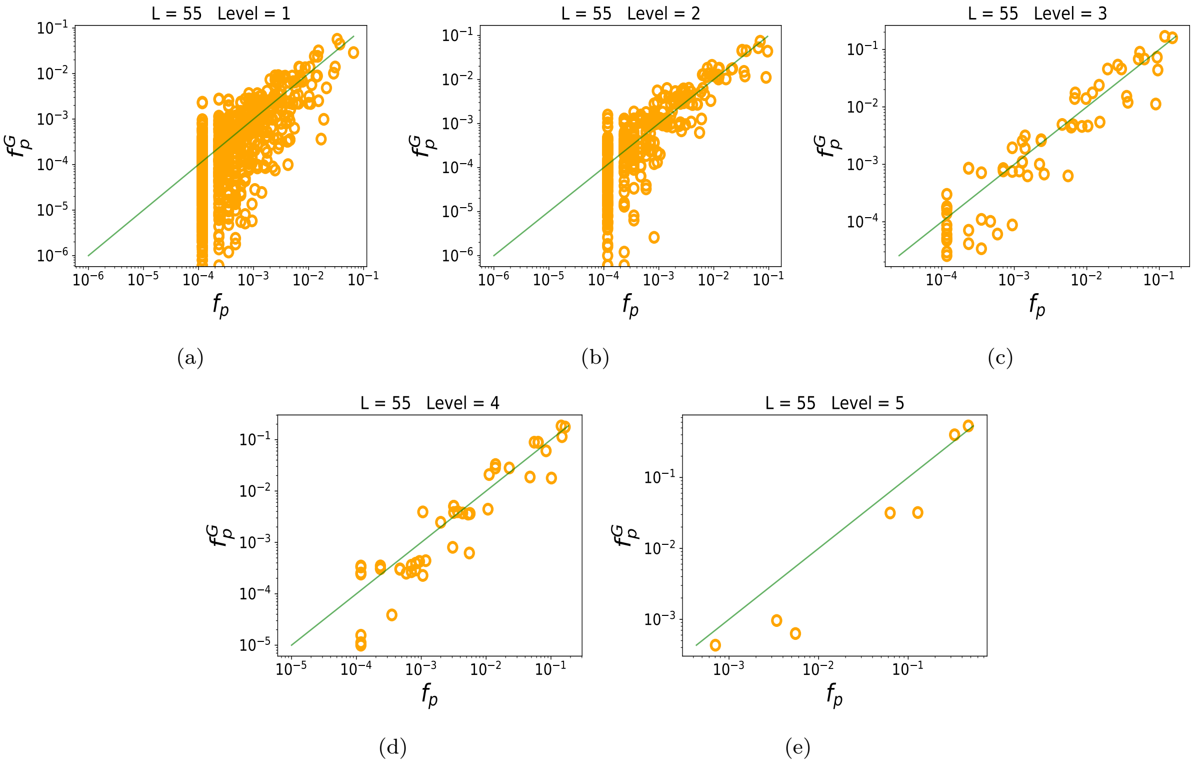


FIG. S2. The frequency of shapes in a database correlates with the frequency in nature for $L = 55$, across all abstraction levels 1, 2, 3, 4 and 5, with $5 \times 10^6$ random samples for each level. For lower abstraction levels, there are fewer samples per shape, and hence more noise. With higher levels and hence more samples per shape, there are less points, but also less noise and a clearer correlation. The green line is simply $x = y$; it is not a fit to the data.

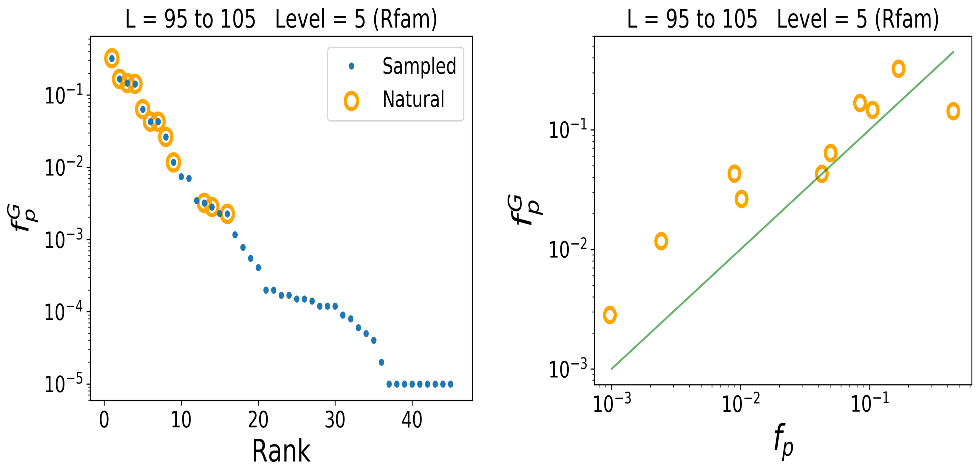


(a)

(b)

FIG. S3. Rank and correlation plots for natural and random data, using Rfam data. (a) Combined data for $L = 95, 96, \ldots, 104, 105$ natural consensus structures rank plot; and (b) $L = 95$ to 105, correlation plot with $r = 0.96$, $p$-value $\approx 10^{-6}$. The data contains 4124 sequences, which yielded 13 unique shapes (level 5).

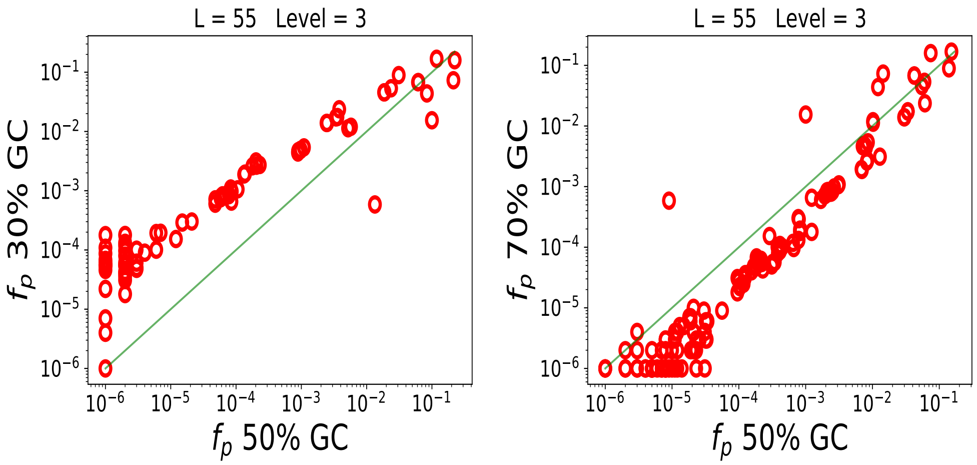


(a)

(b)

FIG. S4. There is a high correlation between the frequencies of RNA shapes when sampling random sequences of different GC contents. (a) 50% GC content vs 30% GC content and (b) 50% vs 70%.

### D. Suboptimal structures

Biologists usually operate with the approximation that there is only one secondary structure for any given RNA sequence. However, if one uses the thermodynamic cost function of a typical folding program, then there are many different RNA structures (hence abstract shapes) which a given sequence can adopt which are within a few $k_BT$ of the minimum free energy (MFE) structure. Thus one can calculate the fraction of time spent in each shape determined by the free energy of each structure via the Boltzmann distribution. Here we investigate these so-called suboptimal structures, by comparing the probability of abstract shapes from sampling, when a single shape is assigned to a sequence, and the probability of a shape if suboptimal shapes are also included. Suboptimal shapes were calculated using the `RNAsubopt` function of the Vienna package, see `www.tbi.univie.ac.at/RNA/RNAsubopt.1.html`.

We study here the correlation between two variables: $f_p$ is the probability of obtaining a given abstract shape on random sampling of a sequence, assuming that only one shape is assigned to each sequence which corresponds to the MFE dot-bracket secondary structure. For example, if a sequence has (((...)))............ as its MFE structure, then the corresponding shape (level 3) would be `[]`.

The second variable $f_p$ *subopt.*, is the probability of obtaining a shape, summing over the Boltzmann probability weights from different sequences. For example if a sequence has (((...)))............ as its MFE structure, then the corresponding shape (level 3) would be `[]`, but at the

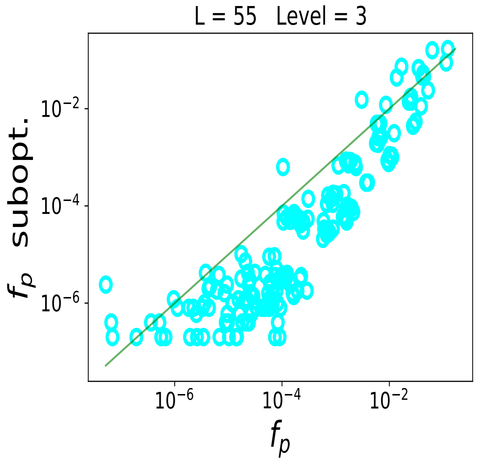


(a)

FIG. S5. There is a high correlation between the frequencies of RNA shapes when using a single shape for each sequence (x-axis), and when incorporating suboptimal structures (y-axis).

same time that sequence would have a certain probability of adopting many different dot bracket structures like eg (((...))).......(((...))) which would have shape `[][]`. Summing the Boltzmann weighted probabilities of the shape `[][]` from different sequences gives the total $f_p$ *subopt.* value for `[][]` etc...

Figure (S5) was obtained using the $L = 55$ level 3 data from the main text ($5 \times 10^6$ samples) to specify $f_p$, and 1000 random sequences with their many respective suboptimal structures and shapes to specify $f_p$ *subopt.*. All suboptimal structures up to 10kT or 6kcal/mol were incorporated for each sequence (note that the number of different suboptimal structures grows very large for large energy gaps, but this is compensated by small probabilities for larger energy gaps).

The correlation in Figure (S5) is high at 0.90 (p-val$< 10^{-6}$) which indicates that whether we study the frequencies of MFE shapes as defined by $f_p$ (as employed in the maintext) or defined by $f_p$ *subopt.*, we would get qualitatively similar results. Nevertheless there are small differences, and these may vary from structure to structure, suggesting that this direction of research is promising for future investigations.

[1] R. Giegerich, B. Voß, and M. Rehmsmeier, Nucleic Acids Research **32**, 4843 (2004).

[2] I. Kalvari, J. Argasinska, N. Quinones-Olvera, E. P. Nawrocki, E. Rivas, S. R. Eddy, A. Bateman, R. D. Finn, and A. I. Petrov, Nucleic acids research **46**, D335 (2018).

[3] I. Kalvari, E. P. Nawrocki, J. Argasinska, N. Quinones-Olvera, R. D. Finn, A. Bateman, and A. I. Petrov, Current protocols in bioinformatics **62**, e51 (2018).

[4] J. Waldispühl and Y. Ponty, in *Research in Computational Molecular Biology*, edited by V. Bafna and S. C. Sahinalp (Springer Berlin Heidelberg, Berlin, Heidelberg, 2011) pp. 501–515.

[5] K. Dingle, S. Schaper, and A. A. Louis, Interface focus **5**, 20150053 (2015).
